# Abacavir Use and Risk for Myocardial Infarction and Cardiovascular Events: Pooled Analysis of Data From Clinical Trials

**DOI:** 10.1093/ofid/ofy086

**Published:** 2018-04-20

**Authors:** Cassandra Nan, Mark Shaefer, Rimgaile Urbaityte, James Oyee, Judy Hopking, Leigh Ragone, Teodora Perger, Beta Win, Harald Vangerow, Cynthia McCoig, Vani Vannappagari

**Affiliations:** 1 Real World Evidence & Epidemiology, GlaxoSmithKline, Stevenage, Hertfordshire, United Kingdom; 2 Global Medical Sciences, ViiV Healthcare, Research Triangle Park, North Carolina; 3 Clinical Statistics, Stockley Park, United Kingdom; 4 Epidemiology & Real World Evidence, ViiV Healthcare, Research Triangle Park, North Carolina; 5 Safety & Pharmacovigilance, ViiV Healthcare, GSK House, United Kingdom; 6 Global Clinical Safety & Pharmacovigilance, Stockley Park, United Kingdom; 7 Safety Evaluation & Risk Management, GlaxoSmithKline, Stockley Park, United Kingdom; 8 Clinical Development, ViiV Healthcare, Tres Cantos, Spain

**Keywords:** abacavir, acute myocardial infarction, angina, cardiovascular event, coronary artery disease, HIV, pooled analysis, safety

## Abstract

**Background:**

Some observational studies and randomized controlled trials (RCTs) have suggested an association between abacavir (ABC) use and myocardial infarction (MI), whereas others have not.

**Methods:**

This pooled analysis of 66 phase II–IV RCTs estimates exposure-adjusted incidence rates (IRs) and relative rates (RRs) of MI and cardiovascular events (CVEs) in participants receiving ABC- and non-ABC-containing combination antiretroviral therapy (cART). The primary analysis of MI included ABC-randomized trials with ≥48-week follow-up. Sensitivity analyses of MI and CVEs included non-ABC-randomized and <48-week follow-up trials.

**Results:**

In 66 clinical trials, 13 119 adults (75% male, aged 18–85 years) were on ABC-containing cART and 7350 were not. Exposure-adjusted IR for MI was 1.5 per 1000 person-years (PY; 95% confidence interval [CI], 0.67–3.34) in the ABC-exposed group and 2.18 per 1000 PY (95% CI, 1.09–4.40) in the unexposed group. The IR for CVEs was 2.9 per 1000 PY (95% CI, 2.09–4.02) in the exposed group and 4.69 per 1000 PY (95% CI, 3.40–6.47) in the unexposed group with studies of ≥48 weeks of follow-up, with an RR of 0.62 (95% CI, 0.39–0.98). The inclusion of nonrandomized and shorter-duration trials did not significantly change the RR for MI or coronary artery disease.

**Conclusions:**

This pooled analysis found comparable IRs for MI and CVEs among ABC-exposed and -unexposed participants, suggesting no increased risk for MI or CVEs following ABC exposure in a clinical trial population. Modifiable risk factors for MI and CVEs should be addressed when prescribing ART.

Cardiovascular disease (CVD) is a leading cause of death in HIV-positive individuals, accounting for approximately 11% of the total deaths in this population [1]. The proportionate mortality due to CVD in HIV-positive individuals in the United States has significantly increased between 1999 and 2013 [2]. The risk of CVD is higher in HIV-positive individuals compared with HIV-negative individuals [3–5]. The reported incidence of myocardial infarction (MI) in cohort studies ranges from 3 to 11 cases per 1000 patient-years in HIV-positive individuals and from 2 to 7 cases per 1000 patient-years in HIV-negative individuals [5–8]. The prevalence of many CVD risk factors tends to be higher among HIV-positive individuals than among HIV-negative individuals, and these factors must be accounted for in any assessment of the relative incidence of CVD [3, 5, 6, 9]. For example, data collected in 2 large US hospitals between 1996 and 2004 found a significantly higher prevalence of smoking (38% vs 18%), hypertension (21% vs 16%), diabetes (12% vs 7%), and dyslipidemia (23% vs 18%) in HIV-positive individuals compared with HIV-negative individuals [5]. Mechanisms for increased CVD risk in HIV remain incompletely defined and probably include both direct and indirect effects of HIV infection. Several studies have reported that HIV-associated inflammation and immune activation play a role in the increased risk of CVD. Furthermore, a recent longitudinal cohort study found that advanced HIV-positive treatment-naïve patients were at even higher risk of CVD, even in the first year after treatment initiation [10]. Additionally, exposure to combination antiretroviral therapy (cART) may play a role in the exacerbation of risk factors for CVD [9, 11, 12]. A recent study did not find any consistent associations between long-term use of cART and subclinical plaque, which increases the risk of CVD. The study concluded that the increased risk of CVD among cART- exposed, HIV-positive individuals cannot be solely attributed to the use of cART [13].

Several studies have suggested an association between abacavir (ABC) use and increased risk of MI [6, 8, 14–19], the first of which was the Data Collection on Adverse Events of Anti-HIV Drugs (D:A:D) study [18], which reported a relative risk of 1.91 (95% confidence interval [CI], 1.50–2.42). This international, multicohort collaboration was specifically set up to prospectively assess the incidence of MI among HIV-positive individuals who received cART. Other studies—including meta-analyses conducted on clinical trial data by GlaxoSmithKline (GSK) [20], the US Food and Drug Administration [21], and independent researchers [22, 23]—have since presented results that do not confirm an association between ABC and increased risk of MI [3, 24–30]. Although strict inclusion and exclusion criteria make RCTs less prone to selection and classification biases and confounding, they may also limit comparability with HIV-infected patients in clinical practice. Additionally, clinical trials were often not designed to detect signals for cardiovascular events (CVEs) and, therefore, may not be sufficiently powered, or events may not have been adjudicated. By contrast, observational studies are subject to channeling bias. It has been previously reported that patients with worse cardiac profiles were prescribed ABC more often than patients with fewer risk factors [18]. Although ABC is not known to adversely affect lipids and glucose metabolism, factors that are normally considered to be pro-atherogenic, a recent systematic review did report some experimental evidence indicating that ABC might induce vascular inflammation. However, the authors did conclude that there is still insufficient evidence in support of or against an association between ABC exposure and cardiovascular outcomes [31].

Since the last analysis of GSK/ViiV Healthcare clinical trial data in 2009 [20], several new GSK/ViiV Healthcare–sponsored clinical trials have been conducted, generating additional data on ABC use and risk for MI and CVEs. While the published evidence remains conflicting, and a plausible biological mechanism for this potential association has not yet been identified, the current pooled analysis aims to estimate the exposure-adjusted incidence rate and relative rate of MI and CVEs reported in individuals treated with ABC-containing and non-ABC-containing cART regimens in ViiV Healthcare–sponsored clinical trials conducted up to December 2016.

## METHODS

Studies were identified through the GSK clinical trial repository. This pooled analysis was based on study reports from all GSK/ViiV Healthcare–sponsored clinical trials in which participants were exposed to ABC, with ≥24 weeks of exposure to cART and for which at least the primary objective had been completed by December 2016. Exposure was defined as being on an ABC-containing regimen or non-ABC-containing regimen as part of study randomization or standard of care. Antiretroviral therapy (ART) taken before study entry was not considered. Each study participant contributed to only 1 of the exposure categories, except in the STRIIVING study, in which participants were allowed to switch after the initial treatment. Study-level data were assessed if participants were randomized to receive ABC. Participant-level data were used if ABC was assigned as standard of care at the discretion of the investigators.

The same definitions as in the initial meta-analysis in 2009 by Brothers et al. [20] were used to select the events of interest based on the MedDRA High-Level Terms of coronary artery disorders not elsewhere classified and ischemic coronary artery disease. MedDRA terms for angina were also included as CVEs, as these were included in the 2009 meta-analysis.

The following specific preferred terms were used for MI: acute myocardial infarction and myocardial infarction. The following specific preferred terms were used for CVE: arteriosclerosis coronary artery, coronary artery disease, coronary artery occlusion, angina pectoris, angina unstable, acute myocardial infarction, myocardial infarction, and myocardial ischemia.

Any preexisting events were not collected as part of the clinical trials unless there was a change in severity during the trial. Therefore, any pretrial MIs and CVEs were not collected and not included in the database.

### Statistical Analyses

All statistical analyses were performed using SAS software, version 9.3 (SAS Institute, Cary, NC). The number of participants, exposure to ABC- or non-ABC-containing regimens in person-years, and number of events were calculated for all studies and summarized according to the exposure category (ABC vs non-ABC). For studies in which participants were randomized to ABC or non-ABC regimens, mean exposure was determined on study level, as opposed to participant-level exposure in trials where ABC was assigned at the investigators’ discretion. The total number of exposed days in each trial was divided by 365.25 to obtain the total exposure in person-years.

Proportions of MIs and CVEs were calculated with their exact binomial 2-sided 95% CIs. A Poisson regression model was fitted to estimate incidence and relative rates of MI and CVEs, which were expressed as rates per 1000 person-years with 95% CIs. The log link was used with an offset, because rates rather than number of events were being modeled. To account for the discontinuation of ABC-containing or non-ABC-containing regimens, incidence rates per 1000 person-years were adjusted for the time participants were exposed to ART during the study period (ie, exposure-adjusted incidence rates). There was no adjustment for confounders. The same summary measures are reported for the main and all sensitivity analyses.

The main analysis included data from studies that were randomized to ABC or non-ABC-containing regimens and included follow-up of ≥48 weeks; 12 studies were included in the previous meta-analysis [20], and 5 GSK-/ViiV Healthcare–sponsored studies were identified post-2009. This analysis was only conducted for MI events. Comparable with the previous analysis [20], this main analysis only included MIs, and the following sensitivity analyses were performed to assess the impact of including studies with shorter follow-up periods and studies in which participants were not necessarily randomized to an ABC-containing regimen. The last 2 sensitivity analyses were also performed for CVEs:

- randomized to ABC or control studies with follow-up durations of <48 weeks conducted for MIs;
- randomized or nonrandomized to ABC or control studies with follow-up durations of ≥48 weeks conducted for MIs and CVEs;
- randomized or nonrandomized studies with follow-up durations of <48 or ≥48 weeks conducted for MIs and CVEs.

## RESULTS

Only studies in which individuals were exposed to ABC-containing cART were eligible for inclusion. A total of 14 studies conducted since the last meta-analysis [20] were included, along with the previous 52 studies, comprising 20 469 study participants in the current analysis. A total of 5959 study participants with ≥48 weeks of follow-up data contributed to the main analysis, of whom 2966 received ABC (3999 person-years) and 2993 did not (3670 person-years). Study participants in all included trials had been on cART for <14 days after receiving a diagnosis of HIV infection, except in ASSURE (EPZ113734) and STRIIVING (201147), in which participants were required to have been on ≥6 months of treatment or even switched regimens. Three studies (ARIES, ASSERT, and HEAT) did not allow participants to have previously taken any nucleoside analog reverse-transcriptase inhibitors and/or non-nucleoside reverse-transcriptase inhibitors and/or protease inhibitors. Table 1 summarizes the included clinical trials since 2009 [20].

**Table 1.** Characteristics of Included Studies Conducted Since 2009^a^

Study Name (Study ID)	Study Period	Phase	Primary Objective	Countries	Male, %	Age Range, Min–Max, y	Study Duration Included in Analysis, wk	ABC-Exposed	ABC-Unexposed
ARIA^b^ (ING117172)	Aug 2013 to Dec 2020	IIIb	To demonstrate the noninferior antiviral activity, safety, and tolerability of DTG/ABC/3TC FDC compared with ATV + RTV and TDF/FTC FDC in HIV-1-infected, ART-naïve women	North America: Canada, United States LATAM: Argentina, Mexico, Puerto Rico Europe: Italy, France, Portugal, Russia, Spain, United Kingdom Africa: South Africa Australasia: Thailand	0	19–79	48	248	247
ARIES (EPZ108859)	Mar 2007 to Jul 2010	IIIb	To compare the safety and efficacy of ATV/r administered QD followed by randomization (1:1) to a simplification regimen of ATV QD or continuation of ATV/r QD, each in combination with ABC/3TC FDC QD in ART-naïve, HIV-1- infected, HLA-B*5701-negative individuals	North America: Canada, United States LATAM: Puerto Rico	85.4	19–72	144	515	0
ASSERT^b^ (CNA109586)	Jun 2007 to Dec 2009	IV	To demonstrate a superior renal safety profile in participants who received ABC/3TC FDC compared with TDF/FTC FDC, both administered with EFV	Europe: Austria, Belgium, Denmark, France, Germany, Ireland, Italy, Latvia, Netherlands, Portugal, Spain, Switzerland, United Kingdom	82.6	18–70	96	192	193
ASSURE^b^ (EPZ113734)	Apr 2010 to Dec 2012	IV	To evaluate the efficacy, safety, and tolerability of the antiviral response between ATV/RTV + TDF/FTC and ATV + ABC/3TC without ritonavir in HIV-1-infected, HLA-B*5701-negative individuals previously suppressed on ATV/RTV + TDF/FTC	North America: United States LATAM: Puerto Rico	79.1	20–68	48	199	97
FLAMINGO (ING114915)	Oct 2011 to Dec 2016	IIIb	To demonstrate the noninferior antiviral activity of DTG 50 mg QD compared with DRV + RTV 800 mg + 100 mg QD, both administered with either ABC/3TC or TDF/FTC, in HIV-1-infected, therapy-naïve participants	North America: United States LATAM: Puerto Rico Europe: France, Germany, Italy, Romania, Russia, Spain, Switzerland	85	18–67	96	159	325
HEAT^b^ (EPZ104057)	Jul 2005 to Apr 2008	IV	To establish that ABC/3TC is virologically noninferior to TDF/FTC when administered in combination with LPV/r in ART-naïve, HIV-1-infected individuals and to compare the safety and tolerability of ABC/3TC vs TDF/FTC when administered with LPV/r	North America: United States LATAM: Puerto Rico	81.8	18–74	96	343	345
LATTE (LAI116482)	Aug 2012 to Dec 2020	IIb	To select a dose of CAB for further evaluation as part of a 2-drug combination ART regimen with rilpivirine, following a 24-wk induction period of CAB with 2 NRTIs (either ABC/3TC or TDF/ FTC) in HIV-1-infected, ART-naïve individuals	North America: Canada, United States	95.9	18–70	24	94	149
LATTE-2 (200056)	Apr 2014 to Dec 2020	IIb	To evaluate a long-acting intramuscular regimen of CAB + RPV for the maintenance of virologic suppression following an induction of virologic suppression on an oral regimen of CAB + ABC/3TC in HIV-1-infected, ART-naïve adults	North America: Canada, United States Europe: Germany, France, Spain	91.3	19–64	48^c^	309	0
MERIT (APV109141)	Mar 2007 to Aug 2008	IIIb	To demonstrate noninferior antiviral activity of FPV/RTV 1400 mg/100 mg QD compared with FPV/RTV 700 mg/100 mg BID, both administered with ABC/3TC FDC QD	Europe: Belgium, France, Germany, Italy, Romania, Russia, Spain, Switzerland, United Kingdom	73.6	18–70	48	212	0
SINGLE^b^ (ING114467)	Feb 2011 to Dec 2015	III	To demonstrate the noninferior antiviral activity of DTG + ABC/3TC QD compared with EFV/TDF/FTC in HIV-1-infected, ART-naïve individuals	North America: Canada, United States Europe: Belgium, Denmark, Italy, France, Germany, Netherlands, Poland, Romania, Spain, United Kingdom Australasia: Australia	84.2	18–85	144	414	419
SPRING-1 (ING112276)	Jul 2009 to Dec 2016	II	To select a DTG QD dose for further evaluation in phase III based on a comparison of antiviral activity and tolerability of a range of oral doses of DTG taken with either ABC/3TC or TDF/FTC in HIV-1-infected, therapy-naïve adults	North America: United States Europe: France, Germany, Italy, Russia, Spain	86.5	20–79	96	67	138
SPRING-2 (ING113086)	Oct 2010 to Jun 2016	III	To demonstrate the antiviral activity of DTG 50 mg QD compared with RAL 400 mg BID, both administered with either ABC/3TC or TDF/FTC in HIV-1-infected, therapy-naïve individuals	North America: Canada, United States Europe: France, Germany, Italy, Russia, Spain, United Kingdom Australasia: Australia	85.6	18–75	96	333	489
STRIIVING^b^ (201147)	Apr 2014 to Dec 2015	IIIb	To compare switching from current ART regimen to ABC/DTG/3TC QD in the treatment of HIV-1-infected, virologically suppressed adults	North America: United States LATAM: Puerto Rico	86.4	22–80	48	519	276^d^
ING116070	Jan 2012 to May 2014	III	A single-arm study of the safety, efficacy, and CNS and plasma PK of DTG 50 mg QD with the ABC/3TC FDC tablet over 96 wk in HIV-1-infected, ART-naïve adults	North America: United States	100	28–52	96	13	0

Abbreviations: ABC, abacavir; ART, antiretroviral therapy; ATV, atazanavir; ATV/r, atazanavir boosted with ritonavir; BID, twice daily; CAB, cabotegravir; CI, confidence interval; CNS, central nervous system; DTG, dolutegravir; EFV, efavirenz; FDC, fixed-dose combination; FPV, fosamprenavir; FTC, emtricitabine; LATAM, Latin America; LPV, lopinavir; LPV/r, lopinavir boosted with ritonavir; NRTI, nucleoside reverse transcriptase inhibitor; PK, pharmacokinetics; QD, once daily; RAL, raltegravir; RPV, rilpivirine; RTV, ritonavir; 3TC, lamivudine; TDF, tenofovir disoproxil fumarate.

^a^For details on studies conducted before 2009, refer to Brothers et al. [20]. Study completion dates are based on the completion of the longest intended follow-up period for all endpoints.

^b^Randomized with respect to ABC-containing therapy.

^c^All study participants (n = 309) took CAB with ≥1 dose of ABC/3TC in the induction phase for 20 wk, and 53 study participants continued taking ABC for an additional 48 wk.

^d^At week 24, individuals originally randomly assigned to current ART switched to ABC/DTG/3TC FDC and were followed for an additional 24 wk (n = 244).

The age range of participants across studies was 18 to 85 years. The majority of study participants were male across studies, except for ARIA in which all participants were female. Table 2 summarizes the demographics and relevant baseline characteristics of participants naïve to and those experienced with treatment.

**Table 2.** Summary of Baseline Demographic and HIV Infection Characteristics of Included Participants

Variable	Treatment-Naïve		Treatment-Experienced	
	ABC-Exposed (n = 2898)	ABC-Unexposed (n = 2306)	ABC-Exposed (n = 718)	ABC-Unexposed (n = 97)
Age at screening, y				
Median (range)	36 (18–79)	36 (18–85)	45 (21–80)	42 (20–68)
Median (IQR)	36 (29–44)	36 (29–44)	45 (36–51)	42 (37–48)
Race/ethnicity, n (%)				
Asian	66 (2.3)	64 (2.8)	18 (2.5)	1 (1.0)
Black	728 (25.1)	541 (23.5)	209 (29.1)	37 (38.1)
American Indian/Alaskan Native	70 (2.4)	69 (3.0)	8 (1.1)	1 (1.0)
White	1970 (68.0)	1569 (68.0)	464 (64.6)	55 (56.7)
Other	64 (2.2)	61 (2.6)	14 (1.9)	3 (3.1)
Missing	0	2 (0.1)	5 (0.7)	0
Sex, n (%)				
Male	2202 (76.0)	1781 (77.2)	603 (84.0)	79 (81.4)
Viral load, log_10_ copies/mL^a^				
Median (range)	4.7 (1.6–7.0)	4.7 (1.7–6.8)	1.6 (1.6–4.1)	1.6 (1.6–3.3)
Median (IQR)	4.7 (4.2–5.2)	4.7 (4.1–5.2)	1.6 (1.6–1.6)	1.6 (1.6–1.6)
CD4 count, cells/mm^3b^				
Median (range)	300 (10–1275)	324 (10–1326)	571 (77–1831)	480 (108–1479)
Median (IQR)	300 (194–421)	324 (222–441)	571 (428–765)	480 (364–651)
CDC category, n (%)				
A: Asymptomatic/lymphadenopathy/acute HIV	2267 (78.2)	1906 (82.7)	514 (71.6)	67 (69.1)
B: Symptomatic, not AIDS	444 (15.3)	271 (11.8)	84 (11.7)	13 (13.4)
C: AIDS	187 (6.5)	129 (5.6)	120 (16.7)	17 (17.5)
Cholesterol, mmol/L^c^				
Median (range)	4.1 (1.3–10.5)	4.1 (1.5–9.8)	4.5 (2.3–8.7)	4.2 (2.7–8.3)
Median (IQR)	4.1 (3.5–4.7)	4.1 (3.5–4.7)	4.5 (4.0–5.2)	4.2 (3.7–5.0)
HDL, mmol/L^d^				
Median (range)	1.0 (0.1–2.9)	1.1 (0.1–3.3)	1.3 (0.4–2.8)	1.2 (0.5–2.7)
Median (IQR)	1.0 (0.9–1.3)	1.1 (0.9–1.3)	1.3 (1.1–1.6)	1.2 (1.0–1.4)
LDL, mmol/L^e^				
Median (range)	2.4 (0.1–8.0)	2.4 (0.0–7.8)	2.5 (0.3–5.7)	2.3 (0.6–5.8)
Median (IQR)	2.4 (1.9–2.9)	2.4 (1.9–2.9)	2.5 (2.0–3.1)	2.3 (1.8–2.8)
Triglycerides, mmol/L^f^				
Median (range)	1.2 (0.3–13.3)	1.2 (0.3–10.9)	1.3 (0.4–11.1)	1.3 (0.5–4.9)
Median (IQR)	1.2 (0.9–1.7)	1.2 (0.8–1.7)	1.3 (0.9–2.0)	1.3 (1.0–2.2)
Glucose, mmol/L^g^				
Median (range)	4.9 (2.3–19.4)	4.8 (1.0–23.3)	5.1 (2.4–22.6)	5.0 (3.9–6.6)
Median (IQR)	4.9 (4.5–5.3)	4.8 (4.5–5.2)	5.1 (4.7–5.5)	5.0 (4.6–5.3)

Baseline laboratory parameters were reported irrespective of fasting state. HDL and LDL were routinely collected in these clinical studies. All studies included ART-naïve participants, except for STRIIVING (201147) and ASSURE (EPZ113734), which included ART-experienced participants.

Abbreviations: ABC, abacavir; ART, antiretroviral therapy; CDC, Centers for Disease Control and Prevention; HDL, high-density lipoprotein; IQR, interquartile range; LDL, low-density lipoprotein.

^a^Treatment-naïve: ABC-exposed, n = 2875. Treatment-experienced: ABC-exposed, n = 709.

^b^Treatment-naïve: ABC-exposed, n = 2875.

^c^Treatment-naïve: ABC-exposed, n = 2638; ABC-unexposed, n = 2094. Treatment-experienced: ABC-exposed, n = 680; ABC-unexposed, n = 92.

^d^Treatment-naïve: ABC-exposed, n = 2636; ABC-unexposed, n = 2094. Treatment-experienced: ABC-exposed, n = 680; ABC-unexposed, n = 92.

^e^Treatment-naïve: ABC-exposed, n = 2591; ABC-unexposed, n = 2068. Treatment-experienced: ABC-exposed, n = 669; ABC-unexposed, n = 91.

^f^Treatment-naïve: ABC-exposed, n = 2636; ABC-unexposed, n = 2095. Treatment-experienced: ABC-exposed, n = 680; ABC-unexposed, n = 92.

^g^Treatment-naïve: ABC-exposed, n = 2681; ABC-unexposed, n = 2161. Treatment-experienced: ABC-exposed, n = 682; ABC-unexposed, n = 92.

Tables 3–5 outline the results from the primary and sensitivity analyses. There were 3 cases of MI and no CVEs in the new trials since the 2009 meta-analysis [20]: 2 cases in the ABC-exposed group (aged 48 and 69 years, onset at days 63 and 92) and 1 in the unexposed group (aged 39 years, onset at day 116). Thus, the total numbers of MIs and CVEs across all included trials were 37 and 74, respectively. The incidence of MI was lower in the ABC-exposed group than in the unexposed group, with a relative rate of 0.69 per 1000 person-years (95% CI, 0.24–1.98). Very similar results were found for CVEs, with a relative rate of 0.62 per 1000 person-years (95% CI, 0.39–0.98). The inclusion of studies with <48 weeks of postexposure follow-up had a very limited impact on the estimates for MIs and CVEs.

**Table 3.** Association Between MIs and ABC Exposure Based on Clinical Trials With ABC Randomization and With ≥48 Weeks of Postexposure Follow-up

	No.	ART Exposure, Person-Years	Events, No.	Proportion of Events (95% CI)	Exposure-Adjusted IR^a^ (95% CI)	RR^a^ (95% CI)
Exposed	2966	3999	6	0.20 (0.07–0.44)	1.50 (0.67–3.34)	0.69 (0.24–1.98)
Unexposed	2993	3670	8	0.27 (0.12–0.53)	2.18 (1.09–4.40)	

^a^Poisson regression model was fitted to estimate IRs and RRs of MIs and cardiovascular events.

Abbreviations: ABC, abacavir; ART, antiretroviral therapy; CI, confidence interval; IR, incidence rate; MI, myocardial infarction; RR, relative rate.

**Table 4.** Association Between MIs and ABC Exposure in Different Scenarios Based on ABC Randomization vs Non–ABC Randomization and Varying Follow-up Lengths

Sensitivity Analysis	ABC Exposure Category	No.	ART Exposure, Person-Years	Events, No.	Proportion of Events (95% CI)	Exposure-Adjusted IR^a^ (95% CI)	RR^a^ (95% CI)
Randomized to ABC, <48 wk follow-up	Exposed	3241	4115	6	0.19 (0.07–0.40)	1.46 (0.66–3.25)	0.69 (0.24–1.99)
	Unexposed	3269	3790	8	0.25 (0.11–0.48)	2.11 (1.06–4.22)	
Randomized or nonrandomized to ABC, ≥48 wk follow-up	Exposed	12 796	12 426	20	0.16 (0.10–0.24)	1.61 (1.04–2.50)	0.79 (0.41–1.53)
	Unexposed	6963	7897	16	0.23 (0.13–0.37)	2.03 (1.24–3.31)	
Randomized or nonrandomized to ABC, <48 or ≥48 wk follow-up	Exposed	13 119	12 520	21	0.16 (0.10–0.25)	1.68 (1.09–2.57)	0.83 (0.44–1.60)
	Unexposed	7074	7956	16	0.23 (0.13–0.37)	2.01 (1.23–3.28)	

^a^Poisson regression model was fitted to estimate IRs and RRs of MIs and cardiovascular events.

Abbreviations: ABC, abacavir; ART, antiretroviral therapy; CI, confidence interval; IR, incidence rate; MI, myocardial infarction; RR, relative rate.

**Table 5.** Association Between Cardiovascular Events and ABC Exposure in Different Scenarios Based on ABC Randomization vs Non–ABC Randomization and Varying Follow-up Lengths

Follow-up	ABC Exposure Category	No.	ART Exposure, Person-Years	Events, No.	Proportion of Events (95% CI)	Exposure-Adjusted IR^a^ (95% CI)	RR^a^ (95% CI)
Randomized or nonrandomized to ABC, ≥48 wk	Exposed	12 796	12426	36	0.28 (0.20–0.39)	2.90 (2.09–4.02)	0.62 (0.39–0.98)
	Unexposed	6963	7897	37	0.53 (0.37–0.73)	4.69 (3.40–6.47)	
Randomized or nonrandomized to ABC, <48 or ≥48 wk	Exposed	13 119	12520	37	0.28 (0.20–0.39)	2.96 (2.14–4.08)	0.64 (0.40–1.00)
	Unexposed	7074	7956	37	0.52 (0.37–0.72)	4.65 (3.37–6.42)	

^a^Poisson regression model was fitted to estimate IRs and RRs of MIs and cardiovascular events.

Abbreviations: ABC, abacavir; ART, antiretroviral thereapy; CI, confidence interval; IR, incidence rate; MI, myocardial infarction; RR, relative rate.

## DISCUSSION

Overall, for ABC-exposed and ABC-unexposed participants, the exposure-adjusted incidence rates of MI and CVEs ranged between 1.46 and 4.65 per 1000 person-years across the different analyses in this study, and they are largely in line with—although slightly lower than—previously reported rates in HIV-positive individuals. Although the MI incidence rates were slightly higher among ABC-unexposed participants, the CIs overlapped with those in ABC-exposed participants, indicating no differences observed between the groups. This result is similar to the conclusion in previous GSK meta-analyses [20]. The incidence rates of CVEs among ABC-unexposed participants were higher than those observed in exposed participants when studies with ≥48 weeks of follow-up were included. However, with the limitations of a pooled analysis that did not adjust for potential confounders and the upper limit of the CI being nearly 1.0, it is possible that no real difference was observed between CVE incidence in the exposed and unexposed groups.

According to the Centers for Disease Control and Prevention, 76% of all HIV-positive adults and adolescents in the United States in 2010 were male [32], which is comparable with the proportion of male participants in the current study. Our analysis included several studies in which ABC was prescribed at the discretion of physicians, thereby resembling a real-world setting more closely. Therefore, the results from this analysis can be considered mainly representative for HIV-positive clinical trial participants in high- and middle-income countries, but it may not necessarily be representative of low-resource countries.

Only a review of the GSK/ViiV Healthcare clinical trial database was performed to identify studies for inclusion. It is possible that published data from non–GSK/ViiV Healthcare–sponsored clinical trials might have provided additional insights. As the clinical trials were not specifically designed to evaluate cardiovascular outcomes, the collection of baseline risk factors for CVEs may not have been incorporated in the original study protocols. Although there was only limited variation in the available cardiovascular risk factors at study entry, cardiac events were likely a stopping criterion in the clinical trials, and therefore none of the trial participants had multiple cardiac events. Additionally, there was no additional adjudication for MI and CVE for the current study. Furthermore, pretreatment events were not recorded, making it impossible to determine whether the event reported during the clinical trials was a recurrence or a new event. Although most trials only included treatment-naïve participants, the definition of treatment naïve varied from not having been on any ART for more than several weeks since diagnosis to not having been on specific ART classes before. It is possible that these other ART exposures may have influenced the risk of CVEs as well [33], but it was not possible to include this in the analysis. Similarly, pretrial exposure to ABC was not recorded in STRIIVING and ASSURE—studies whose inclusion criteria required prior exposure to ART. Time of ABC exposure might have been underestimated for these studies if participants were exposed to ABC prior to their trial enrollment, contributing to potentially greater pooled MI and CVE incidence estimates than would otherwise have been the case. However, a pooled analysis in itself may have underestimated the incidence by using summary data rather than participant-level data to calculate exposure time. The total ART exposure in person-years was defined as average time exposed to the treatment, rather than calculated until time to event or end of study, because CVEs were not the primary outcome of interest in the included studies. Therefore, it was not possible to consider the fact that a participant was no longer at risk of MI or a CVE once the event had already occurred. However, it is unlikely that this would have greatly impacted the results, as there were few MIs and CVEs reported in the included studies. Related to this, the relatively small number of events was a limitation to performing a full statistical analysis to explore the impact of potential confounders. An alternative method to the pooled approach would have been to stratify the analysis by study, but the potential for robust interpretation would be limited regardless of analysis methodology due to the scarcity of data. Finally, ART substitutions (eg, ABC/lamivudine to tenofovir disoproxil fumarate/emtricitabine) were not accounted for.

Based on these limitations of the available data, the current pooled analysis is mainly descriptive in nature, and the results might only be generalizable to clinical trial populations. However, precautionary text regarding CVE risk is included in ABC-related product leaflets, possibly making it more likely that the number of cardiac events outside of clinical trials is limited due to lifestyle advice to patients and potentially fewer ABC prescriptions to patients at high risk for CVEs.

## CONCLUSION

MIs and CVEs were uncommon in GSK/ViiV Healthcare–sponsored clinical trials in which participants were exposed to ABC-containing cART. This analysis found comparable incidence rates for MI and CVEs among ABC-exposed and -unexposed participants, suggesting no increased risk for MI or a CVE following ABC exposure in a clinical trial population. This updated analysis, which includes an additional 3999 person-years of ABC exposure and only 3 additional MIs, continues to corroborate the results from previous meta-analyses of clinical trial data [20, 21]. However, clinicians should continue to prescribe ABC according to the information provided in the package insert, and modifiable risk factors should be considered by clinicians according to treatment guidelines to address patient risk for CVD.
